# Electrolyte handling in the isolated perfused rat kidney: demonstration of vasopressin V2-receptor-dependent calcium reabsorption

**DOI:** 10.1080/03009734.2020.1804496

**Published:** 2020-08-19

**Authors:** Krister Bamberg, Lena William-Olsson, Ulrika Johansson, Anders Arner, Judith Hartleib-Geschwindner, Johan Sällström

**Affiliations:** a Translational Sciences and Experimental Medicines, Research and Early Development, Cardiovascular, Renal and Metabolism, BioPharmaceuticals R&D, AstraZeneca, Gothenburg, Sweden; b Bioscience Renal, Research and Early Development, Cardiovascular, Renal and Metabolism, BioPharmaceuticals R&D, AstraZeneca, Gothenburg, Sweden; c Department of Clinical Sciences Lund, Lund University, Lund, Sweden; d Projects, Research and Early Development, Cardiovascular, Renal and Metabolism, BioPharmaceuticals R&D, AstraZeneca, Gothenburg, Sweden; e Department of Medical Cell Biology, Uppsala University, Uppsala, Sweden; f Department of Physiology and Pharmacology, Karolinska Institutet, Stockholm, Sweden

**Keywords:** AVP, ENaC, kidney, vasopressin

## Abstract

**Background:**

The most profound effect of vasopressin on the kidney is to increase water reabsorption through V_2_-receptor (V_2_R) stimulation, but there are also data suggesting effects on calcium transport. To address this issue, we have established an isolated perfused kidney model with accurate pressure control, to directly study the effects of V_2_R stimulation on kidney function, isolated from systemic effects.

**Methods:**

The role of V_2_R in renal calcium handling was studied in isolated rat kidneys using a new pressure control system that uses a calibration curve to compensate for the internal pressure drop up to the tip of the perfusion cannula.

**Results:**

Kidneys subjected to V_2_R stimulation using desmopressin (DDAVP) displayed stable osmolality and calcium reabsorption throughout the experiment, whereas kidneys not administered DDAVP exhibited a simultaneous fall in urine osmolality and calcium reabsorption. Epithelial sodium channel (ENaC) inhibition using amiloride resulted in a marked increase in potassium reabsorption along with decreased sodium reabsorption.

**Conclusions:**

A stable isolated perfused kidney model with computer-controlled pressure regulation was developed, which retained key physiological functions. The preparation responds to pharmacological inhibition of ENaC channels and activation of V_2_R. Using the model, the dynamic effects of V_2_R stimulation on calcium handling and urine osmolality could be visualised. The study thereby provides evidence for a stimulatory role of V_2_R in renal calcium reabsorption.

## Introduction

The most profound effect of vasopressin (AVP) on the kidney is to increase water reabsorption in the collecting duct mediated through vasopressin V_2_-receptors (V_2_R), but there are also data that indicate effects on calcium transport (1,2). The isolated perfused kidney model (reviewed in (3,4)) offers the advantage that test substances and their impact on kidney function can be studied in a more controlled way than in the intact animal. In the isolated organ model, perfusion pressure can be held constant, thereby minimising confounding secondary effects caused by blood pressure alterations occurring in the intact animal. Furthermore, the concentration of electrolytes and pharmacological agents in the perfusion medium can be precisely controlled. Experiments on isolated kidney preparations can be performed using constant perfusion flow. In such preparations the pressure in the vascular system will change with the vessel tone in the kidney (5,6). Therefore, a pressure control system is used in many preparations in order to maintain a stable arterial pressure in the physiological range (3,7). In principle, these systems have a pressure sensor that is connected to a feedback system that regulates the perfusion pump in order to keep the pressure constant. This introduces several technical challenges. The pressure should be measured at the tip of the perfusion cannula in the renal artery, which requires a dual-lumen cannula. However, given the small size of the artery in mice and rats, it is difficult to place this relatively large cannula in the renal artery. To overcome this, a short section of the aorta can be kept to serve as an adapter for the perfusion cannula (7). Another solution is to measure the pressure anywhere in the perfusion system at the level of the kidney. The problem with this approach is that the pressure fall in the fine perfusion cannula is significant and will be flow-dependent. Consequently, the kidney will be perfused with a different pressure than measured. Thus, obtaining a constant perfusion pressure using a feedback system is associated with non-trivial technical problems. In this report, a new approach for perfusion pressure control is presented. In principle a basal pressure-flow calibration curve is constructed at the start of the experiment. The system can thereby automatically correct the flow and maintain the desired pressure at the tip of the perfusion cannula. Using this system, the stability of the kidney in terms of electrolyte handling, glomerular filtration rate (GFR), and urine production was optimised, whereupon the effects of vasopressin (AVP) on renal calcium handling were studied.

The most well-described hormonal regulator of renal calcium handling is parathyroid hormone (PTH), which promotes active calcium reabsorption (8). The parathyroid gland, discovered in Uppsala in 1877 by Ivar Sandström (9), secretes PTH in response to low serum calcium concentrations. Several lines of evidence demonstrate that also AVP stimulates calcium reabsorption. Earlier studies in Brattleboro rats with diabetes insipidus demonstrated that when long-term administration of the selective V_2_R agonist desmopressin (DDAVP) was ceased, the excretion of calcium increased (1). In a more recent clinical trial, patients with central diabetes insipidus (CDI) and nephrogenic diabetes insipidus (NDI) were challenged with DDAVP administration (2); CDI patients demonstrated a reduced excretion of calcium, whereas no change was observed in the NDI patients. Several channels have been identified for calcium transport along the nephron (10), and mechanistic studies have indicated a stimulatory role of AVP both in the cortical thick ascending limb (cTAL) (11) and the collecting duct (12). The specific effects of AVP on calcium handling in the kidney are, however, not fully understood. The isolated kidney model provides unique possibilities to explore the effects of AVP given that it is possible to precisely control the concentration in the perfusate, which is not possible *in vivo* where systemic variations in hormone levels occur.

## Materials and methods

Male Sprague-Dawley rats (CD® IGS, Charles River, Germany) were used. The animals were anaesthetised using isoflurane, the jugular vein was catheterised, and the abdomen opened via a midline incision. The right kidney was dissected free and the ureter was catheterised using PE10 polyethylene tubing. In order to prevent clotting, a bolus injection of 1500 IU/kg bw heparin (Heparin LEO, 5000 IE/mL, LEO Pharma AB, Malmö, Sweden) was administered via the jugular vein before the kidney was perfused. To avoid leakage of perfusion fluid, branches of the renal artery such as the suprarenal artery were ligated. A blunt needle was introduced into the right renal artery through the mesenteric artery and secured by silk thread, whereupon the perfusion was started. The renal vein was cut open and the kidney removed and placed in the perfusion system, where it was kept throughout the experiment. All experiments were approved by the local animal ethics committee (reference number N23/13).

### Perfusion system

A recirculating perfusion system, modified from a previously described method (13), was used with a constant perfusion pressure (90 mmHg) at 37 °C. All parts of the perfusion system up to the kidney were water-mantled and connected to a constant temperature circulating bath, ensuring constant temperature in the renal parenchyma, which is important given that temperature affects kidney function (14). The kidney was perfused through the renal artery by a computer-controlled peristaltic pump (Minipuls III, Gilson Inc., Middleton, WI, USA). The perfusate was returned to the reservoir by another peristaltic pump (Pumpdrive 5001, Heidolph Instruments GmbH, Schwabach, Germany). The returning perfusate was filtered using a glass filter (Porosity 3 or 4, ROBU Glasfilter-Geraete GmbH, Hattert, Germany) followed by a high-flow syringe filter (Acrodisc 32 mm, 5 µm Supor membrane, Pall Life Sciences, Ann Arbour, MI, USA). Constant perfusion pressure was maintained by using a microcomputer (described below) that automatically adjusts the peristaltic pump. The reservoir containing 200 mL perfusion buffer was constantly gassed with 95% O_2_ and 5% CO_2_. The pH in the reservoir was monitored by an electrode and adjusted to physiological levels (pH 7.40) if necessary by addition of small amounts of HCl (1 mol/L).

The perfusion buffer was a modified Krebs–Henseleit buffer with added sodium pyruvate (0.3 mmol/L), sodium l-lactate (2 mmol/L), alfa-ketoglutarate (1 mmol/L), malate (1 mmol/L), urea (6 mmol/L), and 20 amino acids using a stock solution prepared according to Taft (4). At the day of the experiment, bovine serum albumin (BSA) (bovine serum albumin lyophilised powder ≥96%, Sigma-Aldrich, St. Louis, MO, USA), calcium chloride (2.5 mmol/L), FITC-sinistrin (11.25 mg/L; Fresenius-Kabi, Linz, Austria), DDAVP (400 ng/L; Minirin®, Ferring Läkemedel AB, Sweden) were added.

### Pressure control system

A microcontroller was constructed using the Arduino UNO device (www.arduino.cc). The pressure signal from the transducer is fed into the controller via a bridge amplifier. The pressure sensor was placed at the level of the kidney. The output from the microcontroller determines the speed of the peristaltic pump. A computer programme on the controller was developed that continuously calculates the difference between the desired perfusion pressure and the actual perfusion pressure, and applies a correction to the pump rate using a proportional–integral–derivative (PID) regulator (Arduino PID library) in order to maintain the user-set pressure level. Before an experiment was commenced, a calibration function was activated on the controller which runs the pump through a number of pre-defined flow rates. The pressure at each level was recorded by the controller, which generates a calibration curve, using a second order polynomial fit, that is used in the consecutive experiment. This procedure thus allows for a flow-independent control of the perfusion pressure. Flow data from the experiments were collected and stored using a Powerlab data acquisition system (ADInstruments, Bella Vista, NSW, Australia) connected to a personal computer. Ten-minute mean values of the collected perfusion data were calculated for further analysis.

### Experimental series

Three experimental series were performed encompassing five groups. In the first series, the system was optimised by evaluating the effect of 7.5% versus 6% BSA in the perfusate in eight kidneys per group. Previous studies (13,15) have demonstrated that an elevated albumin concentration contributes positively to stability and reabsorptive capacity of the isolated kidney. The albumin concentration considered most beneficial regarding reabsorption and stability was chosen. The second series was performed in order to study the effect of V_2_R stimulation using DDAVP in six kidneys using 7.5% BSA (using the previously performed 7.5% BSA experimental group as control). Finally, in a third series, the response to the epithelial sodium channel (ENaC) inhibitor amiloride (amiloride hydrochloride hydrate, Sigma-Aldrich, St. Louis, MO, USA; dissolved in water and administered to the fluid reservoir, 5 µmol/L) in the presence or absence of DDAVP was assessed in six kidneys per group.

Urine was collected and the volumes quantified gravimetrically throughout the experiment every 10 min. In the middle of each 10-min period, a sample (0.5 ml) was drawn from the reservoir. Samples of urine and perfusate were analysed for FITC-sinistrin using a spectrophotometer (Paradigm Detection Platform, Beckman Coulter). Electrolytes and glucose were analysed using a blood gas analyser (ABL700, Radiometer, Copenhagen, Denmark; or ABX Pentra 400, Horiba Medical, Kyoto, Japan) and osmolality using a micro osmometer (Model 3 MO or 2020; Advanced Instruments, Norwood, MA, USA).

### Calculations and statistics

All parameters were calculated for each 10-min urine collection period. GFR was calculated as the renal clearance of FITC-sinistrin that has similar properties as inulin but better solubility (16). Fractional reabsorption (FR) for sodium, potassium, calcium, chloride, and glucose was calculated as follows:

(1) $$\mathrm{FR}=\;\frac{\text{Filtered amount}-\text{Excreted amount}}{\text{Filtered amount}}$$

The filtered amount was calculated as GFR multiplied by the perfusate concentration of the electrolyte, whereas the secreted amount was calculated as urine flow multiplied by the urine concentration of the electrolyte. The results are reported as mean values ± standard error of the mean. Single comparisons were performed using Student’s *t* test. Multiple comparisons were performed using two-way ANOVA and, when appropriate, followed by Fisher’s post hoc test. *p* < 0.05 was considered statistically significant. Calculations were performed using R version 3.6.2 (R Foundation for Statistical Computing, Vienna, Austria) and Excel (Microsoft, Redmond, WA, USA).

## Results

### Functional optimisation of the isolated perfused kidney

**Figure 1.**
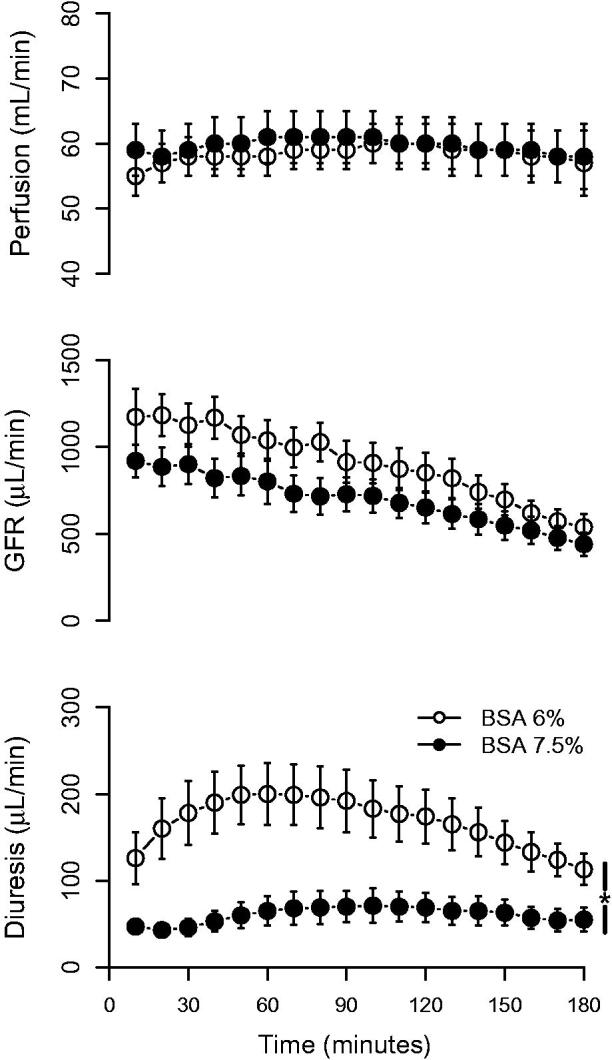
Perfusion, glomerular filtration rate (GFR), and diuresis in kidneys perfused with 6% or 7.5% bovine serum albumin (BSA), respectively. *p < 0.05 between groups, repeated measures ANOVA.

Renal perfusion was stable throughout the experiment and was not affected by altering the albumin concentration (Figure 1). GFR decreased throughout the experiment (approximately 50%) in both groups (Figure 1). When using 7.5% BSA versus 6% BSA, GFR tended to be lower, but more stable over time, but the difference did not reach statistical significance. The diuresis was significantly affected by the albumin concentration; kidneys perfused with 7.5% BSA had a diuresis that was more stable over time throughout the experiment and with less than half the urine output compared with kidneys perfused with 6% BSA (Figure 1). Altering the BSA concentration had a large impact on electrolyte handling (Table 1). The fractional sodium, chloride, calcium, and glucose reabsorption was significantly increased when using 7.5% BSA. Given that 7.5% BSA contributes to a more stable preparation, this concentration was used for the consecutive experimental series.

**Table 1.** Three-hour mean values in kidneys perfused with 6% or 7.5% bovine serum albumin (BSA) or 7.5% BSA + desmopressin (DDAVP; 400 ng/L).

	6% BSA	7.5% BSA	7.5% BSA + DDAVP
Perfusion (mL/min)	58 ± 3	60 ± 4	54 ± 2
GFR (μL/min)	899 ± 105	684 ± 90	747 ± 96
Urine flow (μL/min)	167 ± 30	61 ± 14^a^	27.67 ± 7.96
FR_Na_	0.88 ± 0.02	0.96 ± 0.01^a^	0.97 ± 0.01
FR_K_	0.38 ± 0.02	0.51 ± 0.02^a^	0.61 ± 0.03^b^
Urine Na/K ratio	10.33 ± 1.63	3.61 ± 0.74^a^	3.34 ± 1.07
FR_Cl_	0.86 ± 0.02	0.95 ± 0.01^a^	0.97 ± 0.01
FR_Ca_	0.79 ± 0.02	0.92 ± 0.01^a^	0.99 ± 0.00^b^
FR glucose	0.96 ± 0.01	0.97 ± 0.00^a^	0.98 ± 0.00
Urine osmolality (mOsm/kg)	241 ± 9	230 ± 9	353 ± 9^b^

^a^ *p* < 0.05 versus 6% BSA.

^b^ *p* < 0.05 versus 7.5% BSA.

Ca: calcium; Cl: chloride; FR: fractional reabsorption; GFR: glomerular filtration rate; K: potassium; Na: sodium.

### Effect of desmopressin (DDAVP) on calcium reabsorption

**Figure 2.**
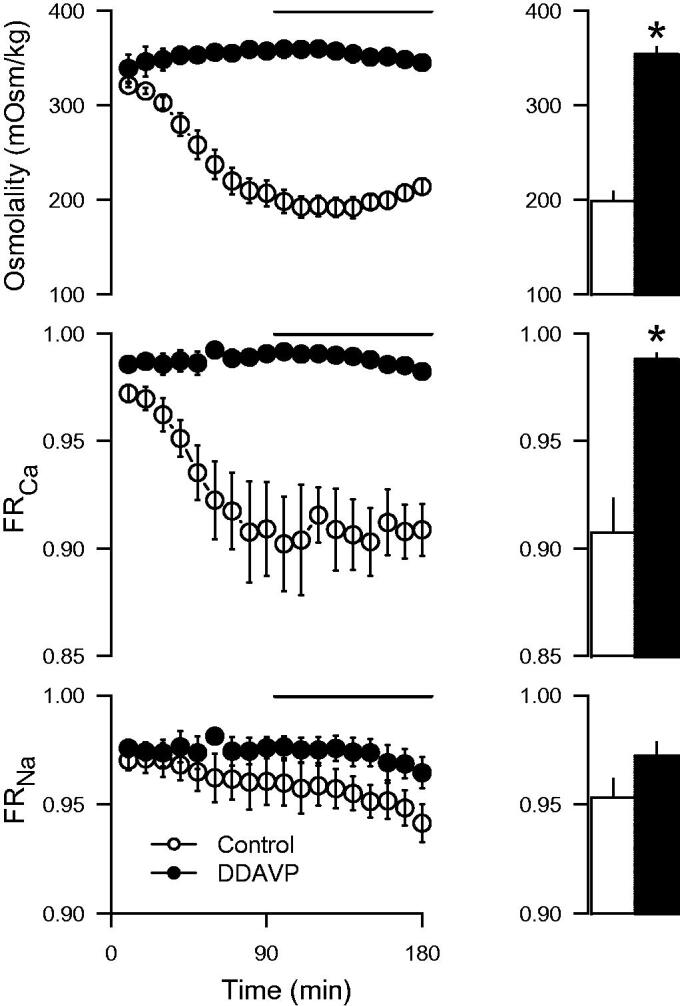
Urine osmolality, fractional calcium (FR_Ca_), and sodium reabsorption (FR_Na_) in kidneys perfused with desmopressin (DDAVP; 400 ng/L) compared to control experiments. The bars represent mean values from the period 90–180 min (indicated by the line). Albumin concentration in the perfusate was 7.5%. *p < 0.05 versus control.

**Figure 3.**
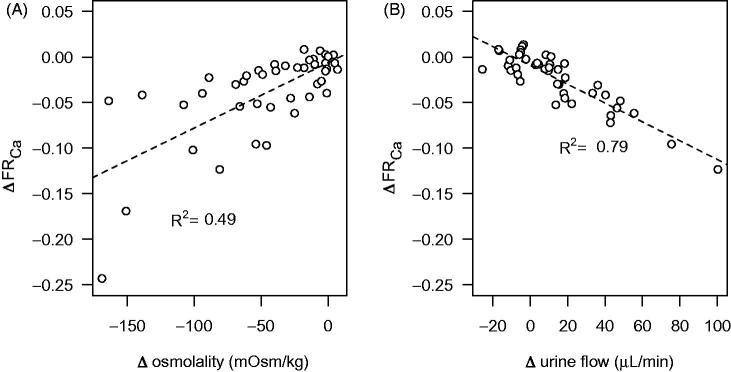
Correlation between change (Δ) in urine osmolality (A) and urine flow (B) and fractional calcium reabsorption (FR_Ca_) in kidneys perfused with 7.5% albumin without DDAVP. Values are calculated as the change from the first 10-min period up to 60 min into the experiment. The values are individual time points derived from the two experimental groups without added DDAVP. A straight line was fitted to the data using linear regression, with the square of the correlation coefficient (*R^2^*) indicated.

In the control group, the urine osmolality gradually fell to about 200 mOsm/kg during the experiment, whereas in DDAVP-treated kidneys the osmolality increased to about 350 mOsm/kg and was sustained at that level (Figure 2). The effect on calcium reabsorption was particularly pronounced and displayed a clear temporal correlation to the changes in urine osmolality, whereas sodium reabsorption was not significantly changed (Figure 2). When the change in osmolality and urine flow up to the first hour of the experiment was plotted against the corresponding change in calcium reabsorption, the reduction in urine osmolality and the increase in urine flow during the experiment was correlated to a reduced calcium reabsorption (Figure 3). The goodness-of-fit of the linear regression was better for urine flow than osmolality. Addition of DDAVP to the perfusate did not affect GFR or renal perfusion compared with perfusate containing only 7.5% BSA but tended to reduce the urine flow rate (Table 1). The DDAVP group exhibited a significantly increased fractional potassium reabsorption, but there was a general tendency for an increase in the fractional reabsorption of all measured electrolytes (Table 1).

### Effect of epithelial sodium channel (ENaC) inhibition

**Figure 4.**
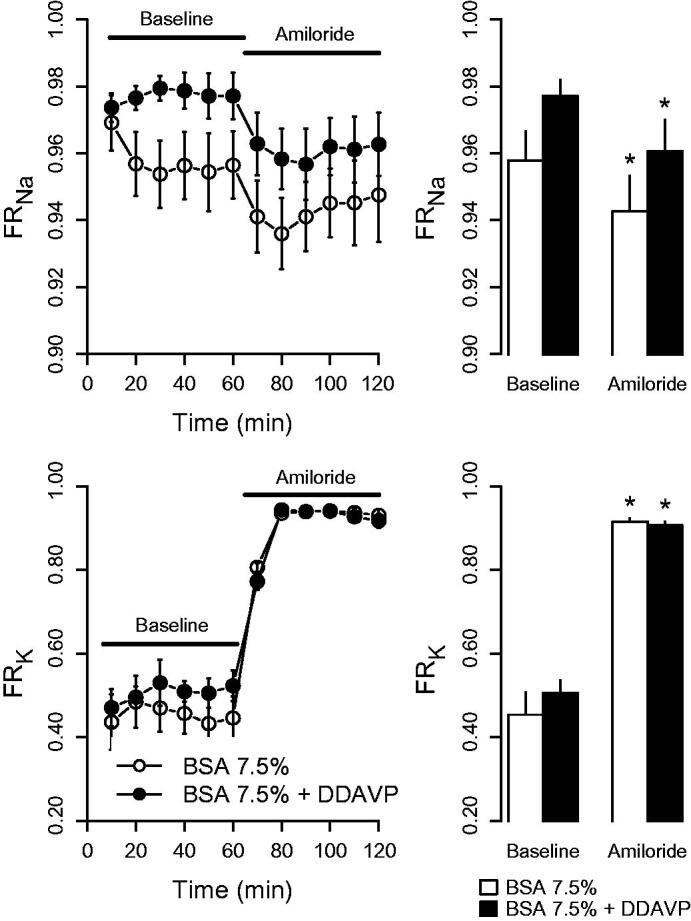
Fractional reabsorption of sodium (FR_Na_) and potassium (FR_K_) in response to amiloride (5 µmol/L) in kidneys perfused with BSA 7.5% or BSA 7.5% + desmopressin (DDAVP; 400 ng/L). The left panel presents the time series data of fractions observed during the 120-min experiment period, whereas the right panel displays mean values at baseline (0–60 min) and at amiloride exposure (60–120 min). *p < 0.05 versus baseline.

Administration of amiloride caused a pronounced increase in fractional potassium reabsorption, whereas fractional sodium reabsorption decreased (Figure 4). The changes were not affected by DDAVP. DDAVP-treated kidneys displayed an elevated fractional calcium reabsorption compared with the control group both during baseline (0.99 ± 0.00 versus 0.94 ± 0.02; *p* < 0.05) and during amiloride treatment (0.99 ± 0.00 versus 0.90 ± 0.02; *p* < 0.05), which agrees with observations in the previous series described above.

## Discussion

The experiments performed demonstrate that the presented new approach for pressure control was able to maintain a functional preparation with stable perfusion for up to 3 h. By using the method, the surgical procedure is facilitated since the cannula can be placed directly in the renal artery; only two experiments failed due to problems with surgery. Using a higher concentration (7.5%) of albumin promoted fluid and electrolyte reabsorption and contributed to a more stable diuresis over time. Addition of DDAVP tended to further promote reabsorption of most electrolytes, leading to an electrolyte reabsorption approaching *in vivo* values (17,18). DDAVP also reversed the fall in urine osmolality that occurred without DDAVP, which further contributed to the stability of the system. The applied experimental model is thus considered suitable for studies on electrolyte transport, which is illustrated by the prompt changes in potassium and sodium handling in response to administration of the potassium-sparing diuretic amiloride that inhibits ENaC. The presented model also demonstrates that it is possible for researchers to design their own experimental equipment fitted to their needs using inexpensive and widely available programmable microcontrollers. Other recently published applications include perfusion controllers for hydrogels (19) and temperature regulators for intravital microscopy (20). We have, however, not found any recent developments regarding perfusion systems for kidneys.

Controlled water reabsorption in the kidney according to physiological needs takes place in the collecting duct where aquaporin-2 (AQP2) channels are incorporated into the apical cell membrane through stimulation of V_2_R by AVP. Aquaporins increase water permeability and consequently increase fluid reabsorption to the hyperosmolar interstitium (21). The effects of AVP on calcium handling is not that well described, but there is evidence of a stimulatory effect on calcium reabsorption from studies both in rodents (1) and in humans (2). In the present experiments, the kidneys are naturally exposed to the normal physiological levels of AVP before the organ was excised from the rat. When perfused without AVP, the antidiuretic effect rapidly declined, as demonstrated by the gradually reduced osmolality and increased urine flow. The reduced osmolality due to absence of AVP is expected, since it is the normal mechanism for regulation of fluid homeostasis in response to water loading and demonstrates that this mechanism is functional in the isolated kidney. The fall in osmolality was accompanied by a matched decrease in calcium reabsorption that, interestingly, had similar temporal characteristics. Given that the effects of vasopressin require aquaporin trafficking (22), the simultaneous effects on water and calcium reabsorption indicate a common tubular target and a related mechanism. Incorporation of aquaporins into the apical membrane involves a complex series of events mediated by the G-protein-coupled V_2_R at the basolateral membrane (reviewed in (22,23)). Among the different signalling pathways involved, calcium signalling has been shown to have a stimulatory role on aquaporin incorporation (24).

The mechanisms for AVP-mediated calcium reabsorption are not clear. Data on PTH-mediated transepithelial calcium transport have been linked to the apical transient receptor potential cation channel subfamily V member 5 and 6 (TRPV5/6) in the distal convoluted tubule and the connecting tubule (25). In the collecting duct, further downstream of the tubular system, canonical transient receptor potential channel member 3 (TRPC3) may instead have a particular role for calcium reabsorption (26). Interestingly, AVP has been shown to induce co-localization of AQP2 channels and TRPC3 cation channels to the apical cell membrane in the medullary collecting duct. Overexpression of these channels in cultured collecting duct cells was also related to increased transepithelial calcium flux (27). Accordingly, calcium channels stimulated by AVP may, besides being involved in the signalling mechanisms for aquaporin trafficking, directly contribute to calcium reabsorption. In line with this, TRPC3 knockout mice displayed an increased urinary calcium concentration compared to their wild-type controls when subjected to water deprivation (28). The correlation observed in the present study between change in osmolality and urine flow and change in calcium reabsorption (Figure 3), as well as their temporal relationship (Figure 2), consequently further supports that AVP increases reabsorption of both water and calcium through incorporation of these channels on the apical membrane of the cells in the collecting duct. The physiological relevance may be to protect against urolithiasis during water restriction, where otherwise calcium levels could be elevated to levels risking precipitation. Another indication on the possible role of this channel in pathophysiology comes from a case report where TRPC3 expression was found to be upregulated in a patient with Williams–Beuren syndrome, a rare neurodevelopmental disorder also associated with hypercalcemia (29). However, TRPC3 expression was found both in the kidney and the intestinal epithelium. Thus, an increased intestinal uptake could also have contributed to the elevated plasma calcium concentrations.

To further assess that the perfused kidney model replicates *in vivo* physiological responses, we assessed the response to ENaC inhibition by amiloride. Administration of amiloride caused a prompt decrease in sodium reabsorption accompanied by an increased potassium reabsorption. These are the expected effects of ENaC inhibition (30), which reduces potassium excretion by hyperpolarizing the principal cells in the collecting duct, thus reducing the electrochemical driving force for potassium extrusion into the lumen. ENaC inhibition will also lower the intracellular sodium concentration, thus reducing basolateral potassium entry through the Na-K-ATPase which also most likely contributes to the reduced potassium secretion (31). Given that both ENaC and TRPC3 are expressed in the principal cells (26,32), the normal response confirms that this nephron segment, where the proposed effects of AVP on calcium reabsorption occurs, is functional in this model. Fractional calcium reabsorption was higher in the DDAVP group both during control conditions and during amiloride treatment. The elevated calcium reabsorption by DDAVP supports the findings in the previous series and also indicates that the acute effect of DDAVP on calcium handling is independent of sodium transport through ENaC.

## Conclusion

In the present report, a new model for pressure control of isolated perfused kidneys has been evaluated and found suitable for studies on electrolyte and fluid transport. The preparation responds to pharmacological inhibition of ENaC channels as well as activation of V_2_R. During V_2_R stimulation, calcium reabsorption was strongly promoted. Using the presented experimental model, the dynamic effects of V_2_R-stimulation on calcium handling and urine osmolality was visualised, which, to the best of our knowledge, has not been previously described. The study thereby provides further evidence for the stimulatory role of V_2_R in calcium transport.
